# Single-cell image analysis reveals a protective role for microglia in glioblastoma

**DOI:** 10.1093/noajnl/vdab031

**Published:** 2021-05-04

**Authors:** Zoe Woolf, Molly E V Swanson, Leon C Smyth, Edward W Mee, Patrick Schweder, Peter Heppner, Bernard J H Kim, Clinton Turner, Robyn L Oldfield, Maurice A Curtis, Richard L M Faull, Emma L Scotter, Thomas I-H Park, Michael Dragunow

**Affiliations:** 1 Department of Pharmacology, Faculty of Medical and Health Sciences, The University of Auckland, Auckland, New Zealand; 2 Centre for Brain Research, Faculty of Medical and Health Sciences, The University of Auckland, Auckland, New Zealand; 3 Department of Anatomy and Medical Imaging, Faculty of Medical and Health Sciences, The University of Auckland, Auckland, New Zealand; 4 School of Biological Sciences, Faculty of Science, The University of Auckland, Auckland, New Zealand; 5 Department of Pathology and Biomedical Science, University of Otago, Christchurch, Christchurch, New Zealand; 6 Department of Neurosurgery, Auckland City Hospital, Auckland, New Zealand; 7 Department of Anatomical Pathology, LabPlus, Auckland City Hospital, Auckland, New Zealand

**Keywords:** immunosuppression, microglia, tumor-associated macrophages, tumor immunology

## Abstract

**Background:**

Microglia and tumor-associated macrophages (TAMs) constitute up to half of the total tumor mass of glioblastomas. Despite these myeloid populations being ontogenetically distinct, they have been largely conflated. Recent single-cell transcriptomic studies have identified genes that distinguish microglia from TAMs. Here we investigated whether the translated proteins of genes enriched in microglial or TAM populations can be used to differentiate these myeloid cells in immunohistochemically stained human glioblastoma tissue.

**Methods:**

Tissue sections from resected low-grade, meningioma, and glioblastoma (grade IV) tumors and epilepsy tissues were immunofluorescently triple-labeled for Iba1 (pan-myeloid marker), CD14 or CD163 (preferential TAM markers), and either P2RY12 or TMEM119 (microglial-specific markers). Using a single-cell-based image analysis pipeline, we quantified the abundance of each marker within single myeloid cells, allowing the identification and analysis of myeloid populations.

**Results:**

P2RY12 and TMEM119 successfully discriminated microglia from TAMs in glioblastoma. In contrast, CD14 and CD163 expression were not restricted to invading TAMs and were upregulated by tumor microglia. Notably, a higher ratio of microglia to TAMs significantly correlated with increased patient survival.

**Conclusions:**

We demonstrate the validity of previously defined microglial-specific genes P2RY12 and TMEM119 as robust discriminators of microglia and TAMs at the protein level in human tissue. Moreover, our data suggest that a higher proportion of microglia may be beneficial for patient survival in glioblastoma. Accordingly, this tissue-based method for myeloid population differentiation could serve as a useful prognostic tool.

**Key Points**

- Single-cell image analysis discriminates microglia from TAMs in glioblastoma.
- A high microglia-to-TAM ratio correlates with increased patient survival in glioblastoma.

**Importance of the Study**

Microglia and macrophages are 2 key myeloid cells that together can comprise up to half the solid mass of glioblastoma tumors. Single-cell RNA sequencing studies have shown that these 2 myeloid populations hold distinct gene profiles and thus potentially differential roles within the tumor microenvironment. Although it is well known that an increase in myeloid-cell load correlates with poor patient prognosis in many tumors, the potentially differing roles of microglia and TAMs on patient survival have yet to be fully defined in human glioblastoma. This study builds on recent transcriptomic studies, showing the successful translation of microglial-specific markers to define and quantify microglia and TAMs in immunofluorescently stained human tissue. Moreover, we show that a higher proportion of microglia to TAMs correlates with increased patient survival in glioblastoma. These findings demonstrate that assessing differential myeloid cell load in glioblastoma tumors could be an additional prognostic tool for assessing patient outcome.

Glioblastoma is the most common and malignant primary brain tumor to affect adults. Despite multimodal treatment approaches, this tumor remains almost invariably fatal, carrying a median survival time of only 15 months.^1^ The microenvironment of glioblastoma tumors is well known to be highly immunosuppressed,^2,3^ with the myeloid compartment of this environment comprised of brain-resident microglia and peripherally invading monocyte-derived macrophages, referred to henceforth as tumor-associated macrophages (TAMs).

Microglia are derived from the yolk-sac mesoderm during early development and migrate into the brain prior to the development of the blood–brain barrier, where they are maintained throughout life through local self-renewal.^4,5,6^ Conversely, TAMs are a population of nonresident macrophages that invade the brain parenchyma from the periphery, originating from fetal liver and bone marrow-derived hematopoietic stem cells.^7–10^ Until recently, microglia and TAMs have been studied as a single, grouped population of “tumor-associated microglia/macrophages,” ^11–13^ this grouping being a consequence of their broadly overlapping gene expression profiles.^14,15^ While these populations share immunological functions including phagocytosis and antigen presentation,^9^ they are ontogenetically distinct and hold unique transcriptomes and epigenetic signatures which may drive differential functions within the tumor microenvironment (TME).^16–18^ Although it has been widely reported that a high myeloid load correlates with poor patient survival in a range of tumors,^19–22^ the independent prognostic capacity of microglia and TAMs is yet to be fully defined.

The emergence of single-cell omics techniques, such as scRNA-seq and mass cytometry, has allowed for a more granular description of immune cell phenotype—identifying microglia and TAM-specific genes both in the normal human brain and in glioblastoma.^18,23–25^ These single-cell omics studies have shown genes such as P2RY12 and TMEM119 are expressed solely by microglia,^26–28^ while genes such as CD14 and CD163 are enriched in TAM populations.^18,19,26,29–31^ Despite studies defining these unique gene profiles, the aforementioned microglial- and TAM-specific markers have yet to be employed at the protein level in human tissue to define myeloid populations.

This study aimed to assess the translatability of these genes to delineate myeloid cell populations in fluorescently stained human brain tissue. We further sought to investigate the correlation between the abundance of tissue-defined microglia and TAMs and patient outcome. To achieve this, we utilized an image analysis pipeline with a flow cytometry-based approach to profile myeloid cell marker expression levels at a single-cell resolution. We demonstrate the successful delineation of microglia from TAMs using P2RY12 and TMEM119, confirming the differential expression of these genes at the protein level. Moreover, we found a higher proportion of microglia to TAMs significantly correlated with increased patient survival in glioblastoma, providing a clinically relevant prognostic factor. This suggests microglia and TAMs may play distinct roles within the glioblastoma TME. Although RNAseq studies have proven fundamental in characterizing these myeloid populations, here we show the translatability of these results to tissue-based staining approaches. We believe this will be essential in facilitating retrospective studies through tissue banks and may serve as a tool for the pathological analysis of patient prognosis.

## Materials and Methods

### Tissue Sources

Human brain tissue was obtained from surgical resection of grade I to grade IV tumors or epilepsy surgeries conducted at Auckland City Hospital. All specimens were collected with written patient consent under the ethical approval from the Northern X Ethics Committee and the University of Auckland Human Participants Ethics Committee (New Zealand). Supplementary Table S1 provides a summary of biopsy information for all tissue. For this study, tumors were grouped into glioblastomas (grade IV), meningiomas (grade I), or low-grade tumors, consisting of grade I–II astrocytomas, schwannomas, and cellular ependymomas. All epilepsy tissues were obtained from temporal lobectomy surgeries for refractory mesial temporal epilepsy with hippocampal sclerosis. In this study, epilepsy tissue served as a quasi-steady-state control and positive control for microglia, with tissue being collected and processed in the same manner as tumor specimens.

### Fluorescent Immunohistochemical Staining

Fresh tumor and epilepsy biopsy tissues were fixed in 15% formalin in 0.1 M phosphate buffer before being paraffin-embedded. 7-µm-thick serial sections were selected for colabeling of Iba1 and CD14 with either P2RY12 or TMEM119, or with Iba1, P2RY12, and CD163. Fluorescent immunohistochemistry was conducted as previously described.^32,33^ Briefly, antigen retrieval with pH 9 tris-EDTA buffer was carried out prior to quenching with TrueBlack Lipofuscin Autofluorescent Quencher (Biotium). All antibodies were diluted in 1% normal donkey serum. Sections were blocked in 10% normal donkey serum and incubated with primary antibodies overnight (Supplementary Table S2).^33–36^ Endogenous peroxidase activity was blocked by incubation with a 50% methanol solution with 1% H_2_O_2_ for 20 min. A biotinylated donkey anti-rabbit secondary antibody (Jackson ImmunoResearch), along with species-specific AlexaFluor-conjugated secondary antibodies (Supplementary Table S2), was incubated at room temperature for 3 h. Tyramide signal amplification of P2RY12 and TMEM119 staining was carried out as previously described.^33^ Sections were counterstained with Hoechst and coverslipped with Prolong Diamond Antifade mountant (Thermofisher Scientific).

Sections were imaged using the MetaSystems VSlide slide scanner (MetaSystems) running Metafer (V. 3.12.1) as previously described.^33^

### Metamorph Custom Journal and Gating Using FCS Express

**Figure 1.**
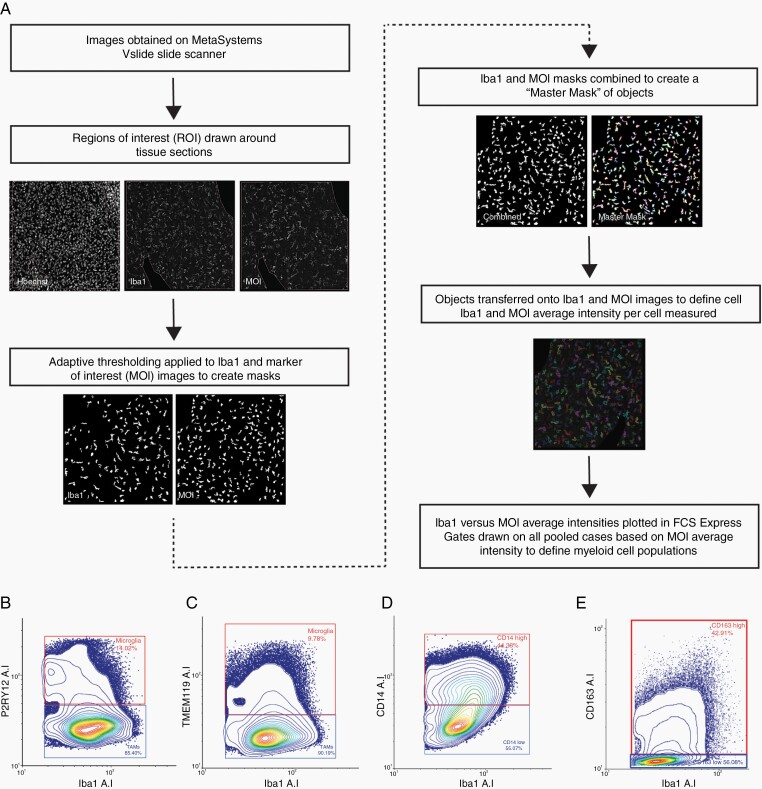
Analysis pipeline summary of MetaMorph custom journal and gating on single-cell average intensity. (A) Cells from all tumor and epilepsy cases were pooled on an XY scatter plot based on Iba1 and marker of interest (MOI) average intensity (P2RY12, TMEM119, CD14, or CD163). Gates were applied to contour plots to define microglia and TAM populations by P2RY12 and TMEM119 average intensity (B and C) and to define CD14^high^ or CD163^high^ and CD14^low^ or CD163^low^ populations (D and E).

We used a previously described single-cell image analysis journal developed in Metamorph software (Molecular Devices)^37^ capable of measuring the single-cell abundances of myeloid cells in immunohistochemically stained human brain tissue (Figure 1). For this study, the journal was adapted to quantify the single-cell expression of Iba1, CD14, or CD163 and one of the microglial-specific markers (P2RY12 or TMEM119) in tumor and epilepsy sections. Briefly, binary masks of Iba1, CD14, or CD163 and microglial-specific marker-positive cells were generated and subsequently combined to form a “master mask,” containing all myeloid cells. Iba1, a pan-myeloid marker acted as our common marker, with CD14, CD163, P2RY12, and TMEM119 defined as markers of interest (MOI). Each object within the master mask was considered a cell, and the average intensities of Iba1, CD14, or CD163 and either P2RY12 or TMEM119 were measured in each cell. Within FCS Express (De Novo Software) single cells were plotted by Iba1 average intensity against MOI average intensity to allow for cell-by-cell population gating and analysis (Figure 1A).

For tumor tissue, population gating was performed on contour plots with the pooled cells from all cases. Based on Iba1 average intensity, all debris were gated out to create a master gate defining all myeloid cells (total cells). Due to inherent differences in the staining patterns between epilepsy and tumor tissue, epilepsy gates were applied separately when gated using P2RY12 and TMEM119 average intensity. Contour plots revealed 2 clear populations when P2RY12 or TMEM119 were plotted against Iba1 (Figure 1B and C). Iba1^+^ P2RY12^+^ and Iba1^+^ TMEM119^+^ cells were gated, and hereafter classified as microglia, while Iba1^+^ P2RY12^−^ and Iba1^+^ TMEM119^−^ cells were gated and classified as TAMs (Figure 1B and C). Contour plots of CD14 or CD163 against Iba1 failed to reveal clear population splits, with a continuum of CD14 or CD163 expression observed (Figure 1D and E). For these markers, gates were applied based on negative staining from epilepsy cases, and manual measurements of cell intensities from raw images to classify 2 populations: CD14 or CD163^high^ and CD14 or CD163^low^ (Figure 1D and E). For population analysis and characterization, abundances of defined cell populations are presented as a proportion of total gated cells. For patient survival analysis, myeloid populations were analyzed as cell densities (total gated cells/mm^2^) to normalize for tissue area.

### Statistical Analysis

Statistical analysis was performed using GraphPad Prism 8 (GraphPad Software Inc.). The normality and variance between the groups were tested using the Shapiro–Wilk normality test and *F*-test of equal variance, respectively. Mann–Whitney *U*-tests were subsequently carried out accordingly when comparing 2 groups. For the comparison of grouped data, two-way analysis of variances with either Sidaks or Tukey’s multiple comparison tests were carried out. All assumptions were tested. Statistical significance was set at *P ≤* .05. All displayed Iba1 and CD14 data analyses were pooled from colabeling with both P2RY12 and TMEM119, with the average of repeated cases used for all statistical analysis and data presentation.

Overall survival was graphed using Kaplan–Meier survival curves using the R software packages *survminer*, *ggplot2*, and *survival*. The single IDH1-mutant glioblastoma patient was excluded from the patient survival analysis. Log-rank analysis was used to compare Kaplan–Meier plots. Exploratory univariate proportional hazards (Cox) regression analysis was conducted on known risk-associated variables to assess their significance on survival. The Akaike Information Criterion model was utilized to determine the variables selected for multivariate proportional hazard (Cox) analysis, which was carried out to investigate the association between the marker expressions and survival. Statistical significance was set at *P* ≤ .05.

## Results

### Microglial-Specific Markers, P2RY12 and TMEM119 Delineate Microglia and TAMs in Immunohistochemically Stained Human Epilepsy and Brain Tumor Tissue

**Figure 2.**
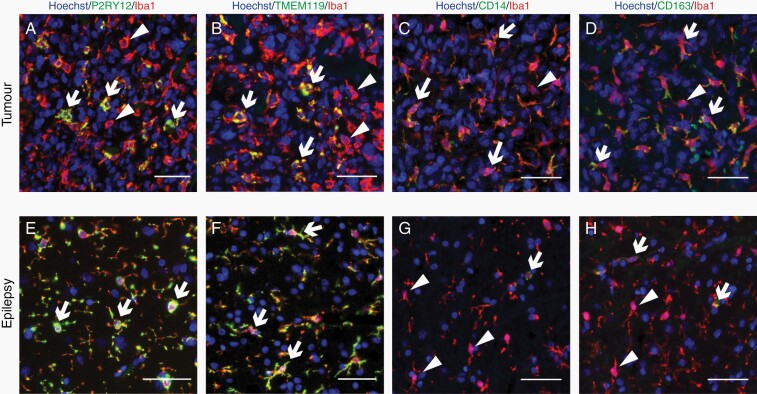
Microglial markers P2RY12 and TMEM119 are present in a subset of tumor myeloid cells. Paraffin-embedded tumor (A–D) and epilepsy (E–H) tissue sections were fluorescently colabeled with pan-marker Iba1, alongside either microglial-specific markers P2RY12 or TMEM119, or proposed TAM-specific markers CD14 or CD163. White arrows denote double-positive cells, while white triangular arrowheads represent single-positive, Iba1-only cells. Scale bars = 50 µm.

To determine the best immunohistochemical markers to distinguish microglia and TAMs in human brain tissue, we colabeled the pan-myeloid cell marker, Iba1, with proposed microglial-specific markers, P2RY12 and TMEM119, or proposed TAM-specific markers, CD14 and CD163 (Figure 2). Both P2RY12 and TMEM119 expression were retained in tumor tissue (Figure 2A and B, E and F). Moreover, when the integrated intensity of P2RY12 and TMEM119 was normalized to myeloid cell density, there was no significant difference in expression between epilepsy and tumor tissue, indicating stable global expression between disease groups (Supplementary Figure S3A and B).

We identified 2 myeloid cell populations following colabeling of Iba1 with proposed microglial-specific markers, P2RY12 and TMEM119: one double-positive microglial population (Iba1^+^ P2RY12^+^ or TMEM119^+^) and one Iba1^+^ only TAM population (Iba1^+^ P2RY12^−^ or TMEM119^−^; Figure 2A–H). While microglia were identified in both tumor and epilepsy cases, TAMs were present almost exclusively in tumor cases (Figure 2A and B, E and F). Colabeling of Iba1 with proposed TAM-specific markers CD14 and CD163 revealed a subset of both Iba1^+^ CD14^+^ or CD163^+^ and Iba1^+^ CD14^−^ or CD163^−^ cells in both tumor and epilepsy tissue (Figure 2C and D, G and H). Although Iba1^+^ CD14^+^ or CD163^+^ cells were identified in epilepsy tissue, where present, they were predominantly associated with blood vessels, indicative of perivascular macrophage expression (Figure 2G and H).

### Accurate Quantification of the Proportion of Microglia to TAMs in Human Brain Tissue by P2RY12 or TMEM119, But Not CD14 or CD163 Immunoreactivity

**Figure 3.**
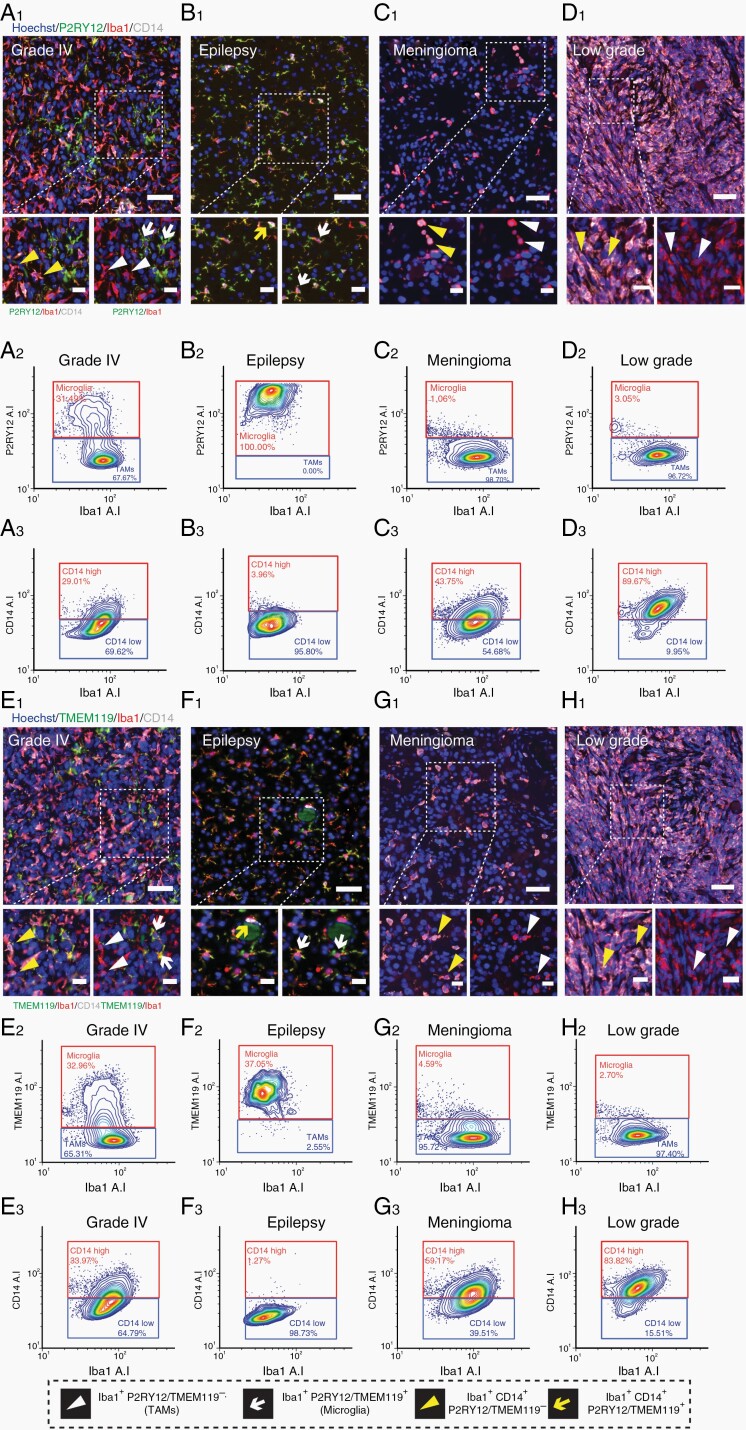
Immunofluorescent identification and gating of microglia and TAM populations using microglial-specific marker expression. Representative immunofluorescent triple labeling using microglial-specific markers P2RY12 with Iba1 and CD14 in grade IV, epilepsy, meningioma, and low-grade tumor tissue (A_1_–D_1_). Cell population identification by cell-specific marker expression is defined in the figure key. Scale bars = 50 µm, 20 µm zoom. Image analysis and cell-by-cell gating using FCS Express for the above representative cases, with P2RY12 (A_2_–D_2_) and CD14 (A_3_–C_3_) plotted against Iba1 average intensity. Representative immunofluorescent triple labeling using microglial-specific markers TMEM119 with Iba1 and CD14 in grade IV, epilepsy, meningioma, and low-grade tumor tissue (E_1_–H_1_). Image analysis and cell-by-cell gating for representative cases, with Iba1 plotted against TMEM119 average intensity (E_2_–H_2_) and CD14 average intensity (E_3_–H_3_).

From our staining (Figure 2), we determined P2RY12 and TMEM119 were the best markers for the delineation of microglia and TAMs and subsequently used these to define populations across epilepsy, meningioma, low-grade, and glioblastoma tumor tissue (Figure 3). Although we observed CD14 and CD163 staining was not restricted to tumor tissue, with expression by Iba1+ cells associated with blood vessels, these markers have previously been described to be more highly expressed by invading macrophages compared to resident brain microglia.^19,25,38^ Therefore, we included these markers in our triple labeling to assess their expression across myeloid-cell populations.

**Figure 4.**
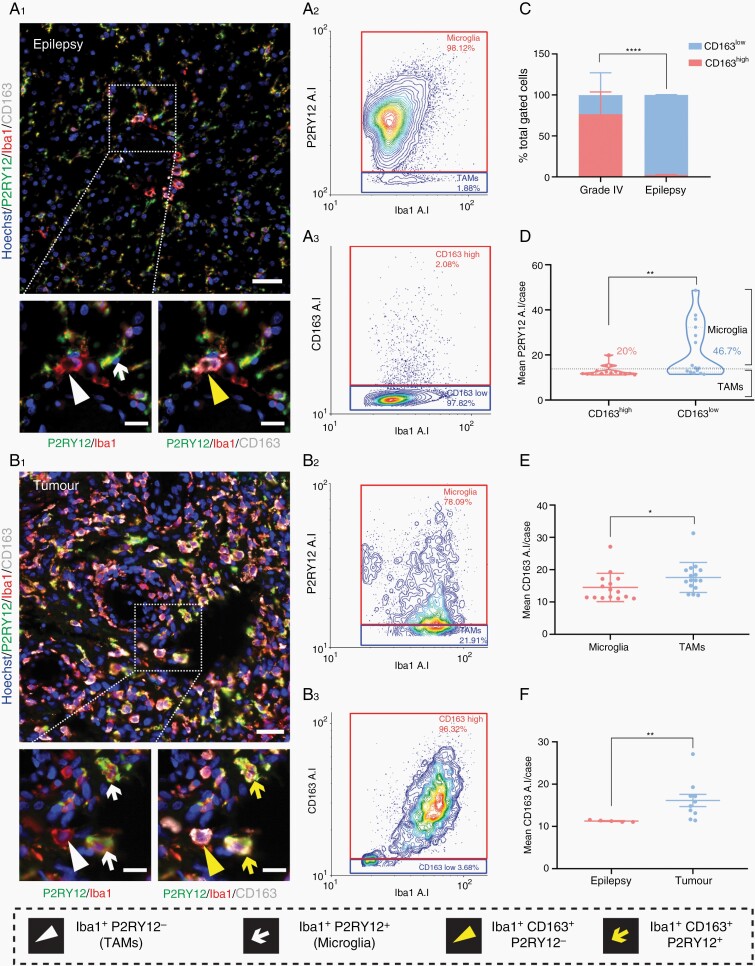
CD163 is not a specific marker for TAMs in human glioblastoma tissue and is upregulated by tumor microglia. Representative immunofluorescent triple labeling with P2RY12, Iba1, and CD163 in epilepsy and grade IV tumor tissue (A_1_, B_1_). Cell population classification by cell-specific marker expression is defined in the figure key. Scale bar 50 µm, 20 µm zoom. Corresponding single-cell resolution gating on Iba1 against P2RY12 or CD163 average intensity to define microglia and TAM populations (A_2_, B_2_), or CD163^high^ and CD163^low^ populations (A_3_, B_3_). Comparison of the mean percentage of CD163^high^ and CD163^low^ cells in grade IV and epilepsy cases using a two-way ANOVA with Tukey’s multiple comparison test (C). Data are presented as mean ± SD, ****P < .0001. Comparison of the mean single-cell average intensity, per case, of P2RY12 for classified CD163^high^ and CD163^low^ cells using a Mann–Whitney test, data are presented as mean ± SD, **P = .0099 (D). Comparison of mean CD163 single-cell average intensity in classified microglia (gated on P2RY12) and TAMs per grade IV tumor case (E). Comparison of mean single-cell CD163 average intensity per case of gated microglia between epilepsy and grade IV tumor tissue (F). Data are presented as mean ± SD; Mann–Whitney test, *P < .0149, **P = .0013.

We developed an automated single-cell image analysis pipeline to identify myeloid populations based on the average staining intensity of the marker of interest (P2RY12, TMEM119, CD14, or CD163). For each tissue type, we have presented representative images of the immunofluorescent staining and subsequent gating of microglia and TAMs based on P2RY12 (Figure 3A1–D1, A2–D2) or TMEM119 intensity (Figure 3E1–H1, E2–H2). While epilepsy tissue was comprised almost solely of ramified microglia, low-grade and meningioma tumor samples contained predominantly TAMs (Figure 3B2–D2, F2–H2). Grade IV cases contained a mixture of both microglia and TAMs (Figure 3A2, E2). CD14 staining was observed variably throughout low-grade, meningioma, and grade IV tumors, but scarcely in epilepsy tissue (Figure 3A1–H1). Subsequent single-cell gating showed that while all tumor subtypes showed a spread of CD14^high^ and CD14^low^ cells, epilepsy tissue was almost completely devoid of CD14^high^ cells (Figure 3A3–D3, E3–H3). Similarly, triple labeling with Iba1, P2RY12, and CD163 revealed that while epilepsy tissue is comprised almost entirely of CD163^low^ cells, grade IV tumor tissue contained varying proportions of CD163^high^ to CD163^low^ cells (Figure 4A3 and B3).

**Figure 5.**
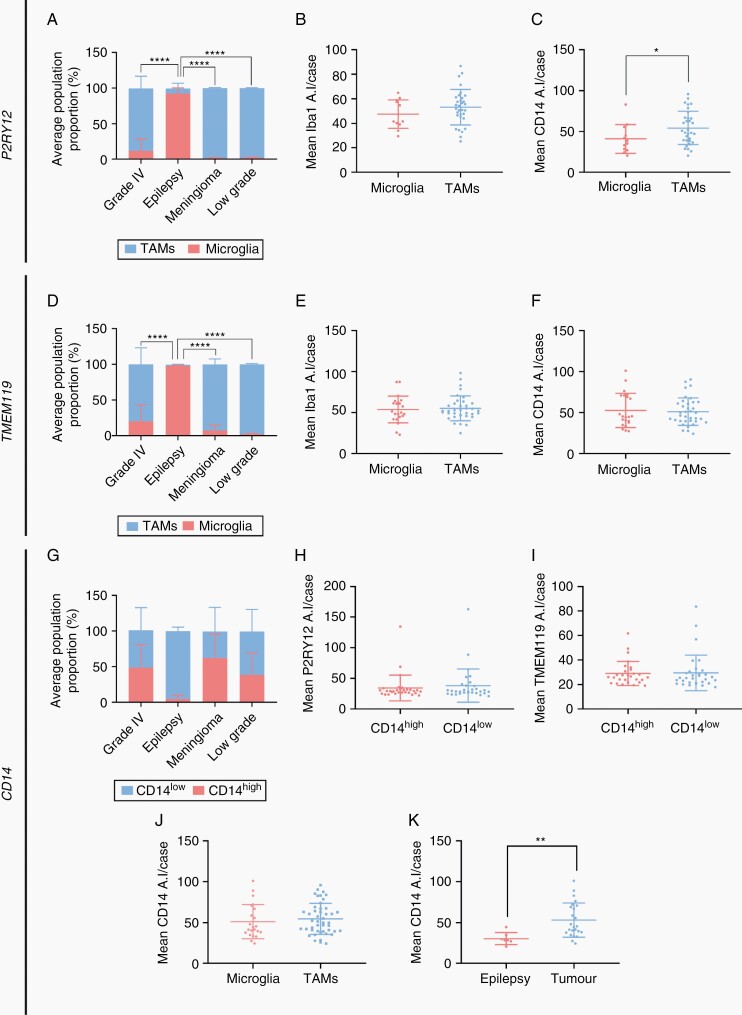
P2RY12 and TMEM119, but not CD14, discriminate 2 myeloid populations in glioblastoma. Using cell-by-cell gating analysis, pooled cells from each case were gated on either P2RY12 (A–C) or TMEM119 average intensity (D–F) and classified as microglia or TAMs, respectively. Pooled cells were also gated based on CD14 average intensity (G–I) to classify cells as CD14^high^ or CD14^low^. Comparison of the mean percentage of microglia and TAMs (or CD14^high^ and CD14^low^ cells) in grade IV, epilepsy, meningioma, and low-grade tumors using a two-way ANOVA with Tukey’s multiple comparison test (A, D, and G). Data are presented as mean ± SD, ****P < .0001. Comparison of the mean single-cell average intensity, per case, of Iba1 and CD14 for classified microglia and TAMs (B and C, E and F), and P2RY12 and TMEM119 for CD14^high^ and CD14^low^ cells (H and I) using a Mann–Whitney test, data are presented as mean ± SD, *P < .05. Comparison of mean CD14 single-cell average intensity per glioblastoma case in gated microglia (pooled P2RY12 and TMEM119) and TAMs (J). Comparison of mean CD14 single-cell average intensity per case of gated microglia between epilepsy and tumor tissue (K). Data are presented as mean ± SD; Mann–Whitney test, **P < .01.

Quantification using cell-by-cell gating allowed for the comparison of the ratio of microglia to TAMs in the 4 brain tissue groups, alongside a comparison of the expression of the MOI within these gated populations (Figure 5A–K). We first assessed the ratio of microglia to TAMs identified from either P2RY12 or TMEM119 immunoreactivity (Figure 5A and D). Pooled analysis of grade IV, meningioma, low-grade, and epilepsy cases revealed significantly higher proportions of microglia to TAMs in epilepsy relative to all other tissue subtypes (Figure 5A and D). While epilepsy cases were comprised almost solely of microglia (Figure 5A and D), both meningioma and low-grade tumor cases contained very low proportions of microglia, with TAMs dominating the myeloid composition (Supplementary Table S3). Grade IV glioblastoma cases showed variable ratios of microglia to TAMs, with pooled analysis revealing a higher proportion of invading TAMs (Supplementary Table S3). To confirm that classification of microglia by either P2RY12 or TMEM119 expression resulted in similar population proportions, a paired *t*-test was carried out on matched tissue cases. No significant difference was found between the microglia-to-TAM ratio, suggesting equivalent delineation across datasets (Supplementary Figure S2A and B).

Given CD14 and CD163 have been previously described as preferential TAM markers, we aimed to detail the immunoreactivity of these markers in defined myeloid populations. As aforementioned, we identified CD14 and CD163 immunoreactivity on both microglia (Iba1^+^ P2RY12^+^ or TMEM119^+^ cells) and TAMs (Iba1^+^ P2RY12^−^ or TMEM119^−^ cells) within tumor tissue (Supplementary Figure S1). Gating and quantification of single-cell staining revealed the proportion of CD14^high^-to-CD14^low^ or CD163^high^-to-CD163^low^cells was variable within tumor tissue (Figures 4B1, C and 5G; Supplementary Table S3). Conversely, epilepsy tissue showed consistently low proportions of CD14^high^ and CD163^high^ cells (Figures 4C and 5G; Supplementary Table S3). Both CD14 and CD163 expression were found to be significantly higher in gated TAM populations (Figures 4E and 5C). We therefore sought to explore the reciprocal relationship, that is, the immunoreactivity of P2RY12 or TMEM119 in gated CD14^high/low^ or CD163^high/low^ populations. The mean single-cell average intensity of these markers reflects the average expression of these proteins within gated populations. No significant difference in the mean single-cell average intensity of P2RY12 or TMEM119 was found between CD14 gated populations (Figure 5H and I). P2RY12 expression was significantly higher in CD163^low^ gated cells, suggesting CD163 is indeed upregulated in TAMs (Figure 4D). However, a proportion of these CD163^high^ populations reached the threshold for microglial classification by P2RY12 average intensity, demonstrating CD163 expression is not limited to TAM populations. To further define CD14 and CD163 immunoreactivity, we then compared the mean single-cell average intensity per case between gated microglia and TAMs. No significant difference in CD14 immunoreactivity was observed, while an increased expression of CD163 was found in gated TAMs (Figures 4E and 5J). Comparison of the mean single-cell CD14 or CD163 average intensity per case revealed an upregulation of both CD14 and CD163 in gated microglia from tumor tissue when compared to epilepsy tissue, showing microglia have the capacity to upregulate these markers in the context of tumors (Figures 4F and 5K).

### Myeloid Cell Populations Define Glioblastoma Patient Survival

Microglia and TAMs are hypothesized to hold different roles in glioblastoma, with the presence of microglia and TAMs within the tumor potentially differentially affecting patient survival.^23^ Therefore, using the quantification of microglia and TAMs based on P2RY12 and TMEM119 immunoreactivity, we investigated potential correlations with patient survival.

**Figure 6.**
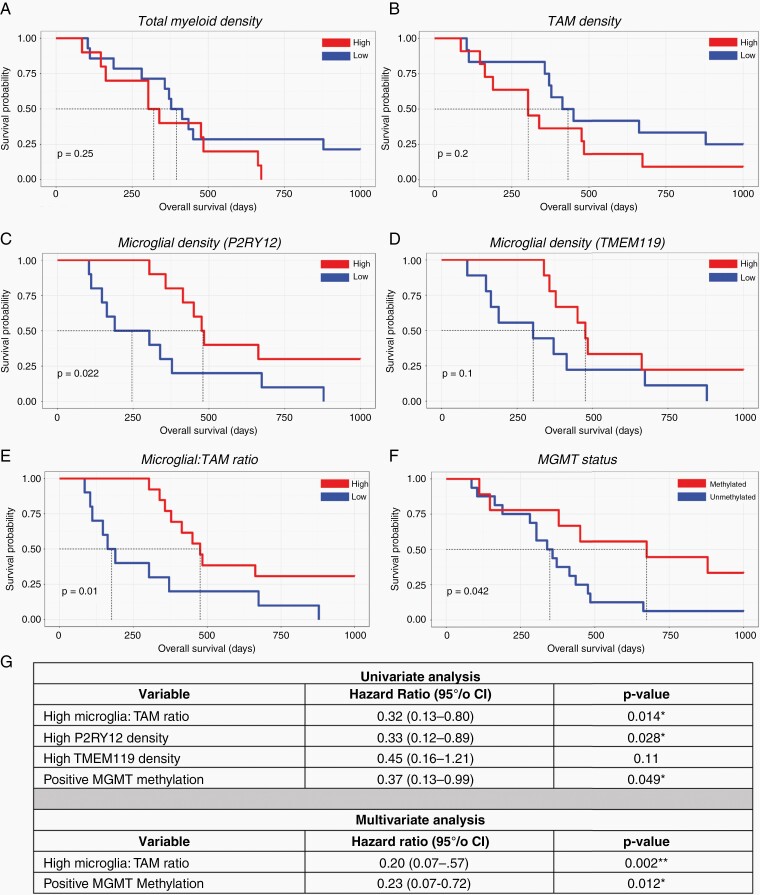
Myeloid cell populations define glioblastoma patient survival. Kaplan–Meier curves exploring the relationship between cell density (cell numbers derived from cell gating/stained area in mm^2^) and patient survival. Total myeloid cell density (total gated cells per case/mm^2^) (A). Total TAM cell density (total gated TAMs per case/mm^2^) (B). Total microglia density (total gated microglia per case/mm^2^) using P2RY12 (C) and TMEM119 expression (D). The ratio of microglia: TAMs per case, utilizing combined P2RY12 and TMEM119 microglial density data (E). MGMT methylation status and patient survival (F). The median value for each variable was used to divide cases into high and low cohort groups. *P < .05. Exploratory univariate proportional hazards (Cox) regression analysis and multivariate analysis of dataset (G); *P < .05, **P < .01.

We first explored the correlation between total myeloid cell density (total gated Iba1^+^ cells/mm^2^) and patient survival. Patients in the high myeloid group showed a shorter overall survival period, although this did not reach significance (Figure 6A). We subsequently sought to determine any potential correlations between the density of TAMs (total gated TAMs/mm^2^) and microglia (total gated microglia/mm^2^) with patient outcome. Patients in the TAM-high cohort showed a shorter survival time relative to the TAM-low cohort, albeit this did not reach significance (Figure 6B). Patients in the microglia-high cohort based on P2RY12 immunoreactivity had significantly longer survival times in glioblastoma, and although a similar trend was observed with TMEM119 microglial density, this was not statistically significant (Figure 6C and D). Reflecting these relationships, when these cell density data were used to determine the ratio of microglia to TAMs within glioblastoma, a higher proportion of microglia to TAMs was seen to correlate with significantly longer survival time (Figure 6E). Furthermore, univariate analysis of the ratio of microglia to TAMs revealed a significant correlation with increased patient survival (*P* = .014, HR = 0.32, Figure 6G). As expected, when split by MGMT methylation status, those in the MGMT-methylated cohort showed significantly increased survival (Figure 6F). Given these findings, we wanted to assess whether a higher microglia-to-TAM ratio still conferred a survival advantage when adjusted for MGMT methylation status. Using a multivariate analysis, we found that when controlled for MGMT methylation status, a higher microglia-to-TAMs ratio still showed a survival advantage (*P =* .002, HR of 0.20, Figure 6G). In addition, positive MGMT methylation status also conferred a survival advantage, independent of the microglia-to-TAMs ratio status (*P =* .012, HR of 0.23, Figure 6G).

## Discussion

This study provides a protein-based validation of microglia–TAM differentiation in human glioblastoma tissue using microglial-specific markers previously highlighted in single-cell transcriptomic studies. Using single-cell immunohistochemical quantification methods, we show that the myeloid compartment of glioblastoma tissue is comprised predominantly of TAMs, and that a higher microglia-to-TAM ratio significantly correlates with longer patient survival. These findings suggest the presence of microglia, rather than TAMs, within the TME may be beneficial for patient outcome. This may serve as a potential prognostic tool and highlights the importance of cell-type-specific therapies, such as those that target peripherally invading TAMs while sparing resident microglia.

We have demonstrated that microglia can be delineated from TAMs in immunohistochemically stained human glioma tissue using the immunoreactivity of microglial-specific markers P2RY12 and TMEM119. This corroborates multiple studies that showed P2RY12 and TMEM119 indeed discriminate microglia from TAMs in rodent RCAS and GL261 models of glioblastoma.^14,24^ In human tissue, although P2RY12 and TMEM119 RNA expression has been shown to vary across different brain malignancies, critically, the cell-type specificity of these markers was found to be maintained.^39^ Here, we show that at the protein level both P2RY12 and TMEM119 expression is maintained across low-grade glioma, meningioma, and glioblastoma tissue, and that the global expression of these proteins does not differ between epilepsy and glioblastoma tissue. Collectively, these data suggest P2RY12 and TMEM119 are robust discriminators of microglia and TAMs in human tissue, both in epilepsy tissue and in the context of glioblastoma.

Using a single-cell image analysis pipeline with the aforementioned markers, we were able to quantify proportions of microglia and TAMs within brain tissue specimens. Concordant with previous studies, we found that epilepsy tissue was comprised almost exclusively of microglia, hence acting as a positive control.^19,39^ In contrast, TAMs were the significant myeloid cell population observed in meningiomas due to the non-brain origin of these tumors.^40^ Indeed, meningioma tissue served as a useful negative control, highlighting the specificity of these microglial markers. While glioblastomas are known to contain both resident microglia and infiltrating TAMs, few studies have methodically assessed the proportions of these cells in human tissue. Here, we show that human glioblastoma is comprised predominantly of invading TAMs, but that there is a high degree of variability between cases—this reflecting the inherent heterogeneity of the TME. To address this heterogeneity, necrotic areas were avoided during analysis; however, due to the nature of tumor surgical resection and tissue processing, it was not possible to standardize the regions of tumor stained.

We were not able to delineate TAMs from microglia using putative TAM-specific markers. A number of markers have been shown to be preferentially upregulated by TAMs, including CD14 and CD163 which were investigated in this study.^19,25,26,29,30,41,42^ In contrast to microglial-specific markers, CD14 and CD163 were expressed on a continuum when plotted against Iba1 average intensity, with no definitive split in intensity values to delineate populations. We found no differential expression of microglial-specific markers between the gated CD14^high/low^ populations, nor was there significant enrichment of CD14 in gated TAMs compared to microglia. Interestingly, CD14 immunoreactivity was not restricted to TAMs and was observed in tumor microglial populations, with a significant upregulation when compared to epilepsy microglia (Figure 5; Supplementary Figure S1). Although CD163 was more highly expressed in the gated TAM populations, similar to the CD14 dataset, this expression was not limited to TAMs, with a proportion of CD163^high^ gated microglia.^33^ Moreover, CD163 was upregulated in tumor microglia when compared to epilepsy microglia. Collectively, these data suggest that in the context of glioblastoma, CD14 and CD163 are not strictly TAM-specific markers and can be expressed by both myeloid cell populations, likely dependent on activation state.

Although it is well established that a high myeloid load correlates with poor patient prognosis in a range of solid tumors,^19–22^ the potentially diverging roles of microglia and TAMs on patient survival have yet to be characterized in human glioblastoma. Previous publications have explored these correlations with varying results dependent on classification by single genes or grouped gene signatures. A recent study utilizing the TCGA-GBM dataset showed that while high CD163 expression correlated with poorer patient survival, when grouped with other genes assigned to the monocyte-derived macrophage signature (CD14, CD68, and MRC1), no significance was found between the low- and high-expressing groups.^19^ Similarly, utilizing the same dataset it has been shown that P2RY12 expression correlates with increased patient survival, although significance was lost following stratification for IDH1 and MGMT status.^43^ These studies have been based on publicly available RNA-seq datasets, highlighting the need to investigate potential correlations between myeloid cell load and patient outcome at the protein level. Critically, immunohistochemical approaches will allow for retrospective studies, and pathological analysis of tissue biopsies, presenting an accessible tool for the analysis of patient prognosis. Using single-cell analysis of in situ fluorescent staining, we identified that a higher proportion of microglia to TAMs correlates with significantly longer survival periods in glioblastoma. Importantly, multivariate analysis revealed this survival advantage was independent of MGMT status. This was further supported by a high microglial load also correlating with increased overall patient survival. Collectively, these data suggest that the presence of more microglia than TAMs within the TME is beneficial for patient survival in glioblastoma. Although these relationships are correlative in nature given the retrospective design of this study, we have demonstrated that characterizing the myeloid composition of tumors through immunohistochemical staining may serve as a useful prognostic tool in assessing patient outcome. Indeed, further studies incorporating additional case numbers will be valuable in determining the prognostic application of these findings.

Recent publications have suggested microglia and TAMs may hold different functional roles within the tumor,^19,23^ lending support to the causal patient outcomes observed in the current study. Microglia have been shown to express a more proinvasive transcriptional profile, possibly participating in the early recruitment and trafficking of immune cells to the tumor, while TAMs adopt a more immunosuppressive transcriptional profile, contributing to the anti-inflammatory milieu.^24,44^ A possible mechanism for these differences may stem from the susceptibility of these myeloid populations to the immunosuppressive signals within the tumor. As TAMs enter the tumor as monocytes before differentiation into macrophages and predominantly reside in the tumor core,^19,45^ these cells may be more susceptible to tumor-induced phenotypic differentiation. Alternatively, these differences may simply stem from distinct transcriptomes and epigenetic signatures.

In this study, we demonstrate the translatability of microglial-specific genes P2RY12 and TMEM119 in robustly discriminating microglia and TAMs in fluorescently stained human glioblastoma tissue. Moreover, our data reveal a higher proportion of microglia to TAMs positively correlates with patient survival in glioblastoma, presenting as an accessible method to aid in the evaluation of patient prognosis. This adds to the current consensus in the field, suggesting microglia and TAMs may indeed play differential roles in glioblastoma. Although beyond the scope of this study, future research utilizing single-cell profiling techniques such as multicolor flow cytometry will be essential in uncovering the mechanisms by which microglia and TAMs differentially affect patient outcome. Such studies should combine the aforementioned cell identification markers with functional markers to define the mechanistic roles of these cells within the TME. Understanding how these myeloid populations individually affect glioblastoma development and progression will be central in the future development of targeted therapies.

## Funding

We acknowledge the following funding bodies for their support of this research: Programme Grant from the Health Research Council of New Zealand, the Hugh Green Foundation, the Gooduck Charitable Trust, the Auckland Medical Research Foundation, the New Zealand Neurological Foundation, and the Douglas Charitable Trust.

## Supplementary Material

vdab031_suppl_Supplementary_Figure_S1

vdab031_suppl_Supplementary_Figure_S2

vdab031_suppl_Supplementary_Figure_S3

vdab031_suppl_Supplementary_Table_S1

vdab031_suppl_Supplementary_Table_S2

vdab031_suppl_Supplementary_Table_S3

vdab031_suppl_Supplementary_Materials

## References

[1] Wen PY , Kesari S. Malignant gliomas in adults. N Engl J Med. 2008;359(5):492–507. 18669428 10.1056/NEJMra0708126

[2] Watters JJ , Schartner JM, Badie B. Microglia function in brain tumors. J Neurosci Res. 2005;81(3):447–455. 15959903 10.1002/jnr.20485

[3] Chen Z , Hambardzumyan D. Immune microenvironment in glioblastoma subtypes. Front Immunol. 2018;9:1004. 29867979 10.3389/fimmu.2018.01004 PMC5951930

[4] Yin Y , Qiu S, Li X, Huang B, Xu Y, Peng Y. EZH2 suppression in glioblastoma shifts microglia toward M1 phenotype in tumor microenvironment. J Neuroinflammation. 2017;14(1):220. 29132376 10.1186/s12974-017-0993-4 PMC5684749

[5] Ginhoux F , Greter M, Leboeuf M, et al. Fate mapping analysis reveals that adult microglia derive from primitive macrophages. Science. 2010;330(6005):841–845. 20966214 10.1126/science.1194637 PMC3719181

[6] Ajami B , Bennett JL, Krieger C, Tetzlaff W, Rossi FM. Local self-renewal can sustain CNS microglia maintenance and function throughout adult life. Nat Neurosci. 2007;10(12):1538–1543. 18026097 10.1038/nn2014

[7] Chanmee T , Ontong P, Konno K, Itano N. Tumor-associated macrophages as major players in the tumor microenvironment. Cancers (Basel). 2014;6(3):1670–1690. 25125485 10.3390/cancers6031670 PMC4190561

[8] Graeber MB , Scheithauer BW, Kreutzberg GW. Microglia in brain tumors. Glia. 2002;40(2):252–259. 12379912 10.1002/glia.10147

[9] Choi J , Mai N, Jackson C, Belcaid Z, Lim M. It takes two: potential therapies and insights involving microglia and macrophages in glioblastoma. Neuroimmunol Neuroinflamm. 2018;5(10):42.

[10] Liu Y , Cao X. The origin and function of tumor-associated macrophages. Cell Mol Immunol. 2015;12(1):1–4. 25220733 10.1038/cmi.2014.83 PMC4654376

[11] Sørensen MD , Dahlrot RH, Boldt HB, Hansen S, Kristensen BW. Tumour-associated microglia/macrophages predict poor prognosis in high-grade gliomas and correlate with an aggressive tumour subtype. Neuropathol Appl Neurobiol. 2018;44(2):185–206. 28767130 10.1111/nan.12428

[12] Pires-Afonso Y , Niclou SP, Michelucci A. Revealing and harnessing tumour-associated microglia/macrophage heterogeneity in glioblastoma. Int J Mol Sci. 2020;21(3):689. 10.3390/ijms21030689 PMC7037936 31973030

[13] Roesch S , Rapp C, Dettling S, Herold-Mende C. When immune cells turn bad-tumor-associated microglia/macrophages in glioma. Int J Mol Sci. 2018;19(2):436. 10.3390/ijms19020436 PMC5855658 29389898

[14] Haage V , Semtner M, Vidal RO, et al. Comprehensive gene expression meta-analysis identifies signature genes that distinguish microglia from peripheral monocytes/macrophages in health and glioma. Acta Neuropathol Commun. 2019;7(1):20. 30764877 10.1186/s40478-019-0665-y PMC6376799

[15] Wei J , Chen P, Gupta P, et al. Immune biology of glioma-associated macrophages and microglia: functional and therapeutic implications. Neuro Oncol. 2019;22(2):180–194. 10.1093/neuonc/noz212 PMC7442334 31679017

[16] Li Q , Barres BA. Microglia and macrophages in brain homeostasis and disease. Nat Rev Immunol. 2018;18(4):225–242. 29151590 10.1038/nri.2017.125

[17] Utz SG , See P, Mildenberger W, et al. Early fate defines microglia and non-parenchymal brain macrophage development. Cell. 2020;181(3):557–573.e18. 32259484 10.1016/j.cell.2020.03.021

[18] Bowman RL , Klemm F, Akkari L, et al. Macrophage ontogeny underlies differences in tumor-specific education in brain malignancies. Cell Rep. 2016;17(9):2445–2459. 27840052 10.1016/j.celrep.2016.10.052 PMC5450644

[19] Friebel E , Kapolou K, Unger S, et al. Single-cell mapping of human brain cancer reveals tumor-specific instruction of tissue-invading leukocytes. Cell. 2020;181(7):1626–1642.e20. 32470397 10.1016/j.cell.2020.04.055

[20] Mantovani A , Marchesi F, Malesci A, Laghi L, Allavena P. Tumour-associated macrophages as treatment targets in oncology. Nat Rev Clin Oncol. 2017;14(7):399–416. 28117416 10.1038/nrclinonc.2016.217 PMC5480600

[21] Alban TJ , Alvarado AG, Sorensen MD, et al. Global immune fingerprinting in glioblastoma patient peripheral blood reveals immune-suppression signatures associated with prognosis. JCI Insight. 2018;3(21): e122264. 10.1172/jci.insight.122264 PMC6238746 30385717

[22] Bingle L , Brown NJ, Lewis CE. The role of tumour-associated macrophages in tumour progression: implications for new anticancer therapies. J Pathol. 2002;196(3):254–265. 11857487 10.1002/path.1027

[23] Müller S , Kohanbash G, Liu SJ, et al. Single-cell profiling of human gliomas reveals macrophage ontogeny as a basis for regional differences in macrophage activation in the tumor microenvironment. Genome Biol. 2017;18(1):234. 29262845 10.1186/s13059-017-1362-4 PMC5738907

[24] Ochocka N , Segit P, Walentynowicz KA, et al. Single-cell RNA sequencing reveals functional heterogeneity and sex differences of glioma-associated brain macrophages. Nat Commun. 2021;12(1):1151. 10.1038/s41467-021-21407-w PMC7895824 33608526

[25] Darmanis S , Sloan SA, Croote D, et al. Single-cell RNA-Seq analysis of infiltrating neoplastic cells at the migrating front of human glioblastoma. Cell Rep. 2017;21(5):1399–1410. 29091775 10.1016/j.celrep.2017.10.030 PMC5810554

[26] Bennett ML , Bennett FC, Liddelow SA, et al. New tools for studying microglia in the mouse and human CNS. Proc Natl Acad Sci U S A. 2016;113(12):E1738–E1746. 26884166 10.1073/pnas.1525528113 PMC4812770

[27] Butovsky O , Jedrychowski MP, Moore CS, et al. Identification of a unique TGF-β-dependent molecular and functional signature in microglia. Nat Neurosci. 2014;17(1):131–143. 24316888 10.1038/nn.3599 PMC4066672

[28] Buttgereit A , Lelios I, Yu X, et al. Sall1 is a transcriptional regulator defining microglia identity and function. Nat Immunol. 2016;17(12):1397–1406. 27776109 10.1038/ni.3585

[29] Chen Y , Song Y, Du W, Gong L, Chang H, Zou Z. Tumor-associated macrophages: an accomplice in solid tumor progression. J Biomed Sci. 2019;26(1):78. 31629410 10.1186/s12929-019-0568-z PMC6800990

[30] Gabrusiewicz K , Rodriguez B, Wei J, et al. Glioblastoma-infiltrated innate immune cells resemble M0 macrophage phenotype. JCI Insight. 2016;1(2):e85841. 10.1172/jci.insight.85841 PMC4784261 26973881

[31] Sankowski R , Böttcher C, Masuda T, et al. Mapping microglia states in the human brain through the integration of high-dimensional techniques. Nat Neurosci. 2019;22(12):2098–2110. 31740814 10.1038/s41593-019-0532-y

[32] Waldvogel HJ , Curtis MA, Baer K, Rees MI, Faull RL. Immunohistochemical staining of post-mortem adult human brain sections. Nat Protoc. 2006;1(6):2719–2732. 17406528 10.1038/nprot.2006.354

[33] Swanson MEV , Murray HC, Ryan B, Faull RLM, Dragunow M, Curtis MA. Quantitative immunohistochemical analysis of myeloid cell marker expression in human cortex captures microglia heterogeneity with anatomical context. Sci Rep. 2020;10(1):11693. 32678124 10.1038/s41598-020-68086-z PMC7366669

[34] Bennett FC , Bennett ML, Yaqoob F, et al. A combination of ontogeny and CNS environment establishes microglial identity. Neuron. 2018;98(6):1170–1183.e1178. 29861285 10.1016/j.neuron.2018.05.014 PMC6023731

[35] Yu F , Gong L, Mo Z, et al. Programmed death ligand-1, tumor infiltrating lymphocytes and HLA expression in Chinese extrahepatic cholangiocarcinoma patients: possible immunotherapy implications. Biosci Trends. 2019;13(1):58–69. 30773525 10.5582/bst.2019.01003

[36] Chen SJ , Zhang QB, Zeng LJ, et al. Distribution and clinical significance of tumour-associated macrophages in pancreatic ductal adenocarcinoma: a retrospective analysis in China. Curr Oncol. 2015;22(1):e11–e19. 25684992 10.3747/co.22.2150 PMC4324348

[37] Swanson MEV , Scotter EL, Smyth LCD, et al. Identification of a dysfunctional microglial population in human Alzheimer’s disease cortex using novel single-cell histology image analysis. Acta Neuropathol Commun. 2020;8(1):170. 33081847 10.1186/s40478-020-01047-9 PMC7576851

[38] Böttcher C , Schlickeiser S, Sneeboer MAM, et al. Human microglia regional heterogeneity and phenotypes determined by multiplexed single-cell mass cytometry. Nat Neurosci. 2019;22(1):78–90. 30559476 10.1038/s41593-018-0290-2

[39] Klemm F , Maas RR, Bowman RL, et al. Interrogation of the microenvironmental landscape in brain tumors reveals disease-specific alterations of immune cells. Cell. 2020;181(7):1643–1660.e17. 32470396 10.1016/j.cell.2020.05.007 PMC8558904

[40] Guadagno E , Presta I, Maisano D, et al. Role of macrophages in brain tumor growth and progression. Int J Mol Sci. 2018;19(4): 1005. 10.3390/ijms19041005 PMC5979398 29584702

[41] Vidyarthi A , Agnihotri T, Khan N, et al. Predominance of M2 macrophages in gliomas leads to the suppression of local and systemic immunity. Cancer Immunol Immunother. 2019;68(12):1995–2004. 31690954 10.1007/s00262-019-02423-8 PMC11028103

[42] Liu S , Zhang C, Maimela NR, et al. Molecular and clinical characterization of CD163 expression via large-scale analysis in glioma. Oncoimmunology. 2019;8(7):e1601478. 10.1080/2162402X.2019.1601478 PMC6527268 31143523

[43] Zhu C , Kros JM, van der Weiden M, Zheng P, Cheng C, Mustafa DA. Expression site of P2RY12 in residential microglial cells in astrocytomas correlates with M1 and M2 marker expression and tumor grade. Acta Neuropathol Commun. 2017;5(1):4. 28073370 10.1186/s40478-016-0405-5 PMC5223388

[44] Poh AR , Ernst M. Targeting macrophages in cancer: from bench to bedside. Front Oncol. 2018;8:49. 29594035 10.3389/fonc.2018.00049 PMC5858529

[45] Chen Z , Feng X, Herting CJ, et al. Cellular and molecular identity of tumor-associated macrophages in Glioblastoma. Cancer Res. 2017;77(9):2266–2278. 28235764 10.1158/0008-5472.CAN-16-2310 PMC5741820

