# Supplementary figures and images for: Single-cell image analysis reveals a protective role for microglia in glioblastoma
Source: Neurooncol Adv. 2021 May 4;3(1):vdab031. doi: 10.1093/noajnl/vdab031 (PMC8284623; doi:10.1093/noajnl/vdab031)

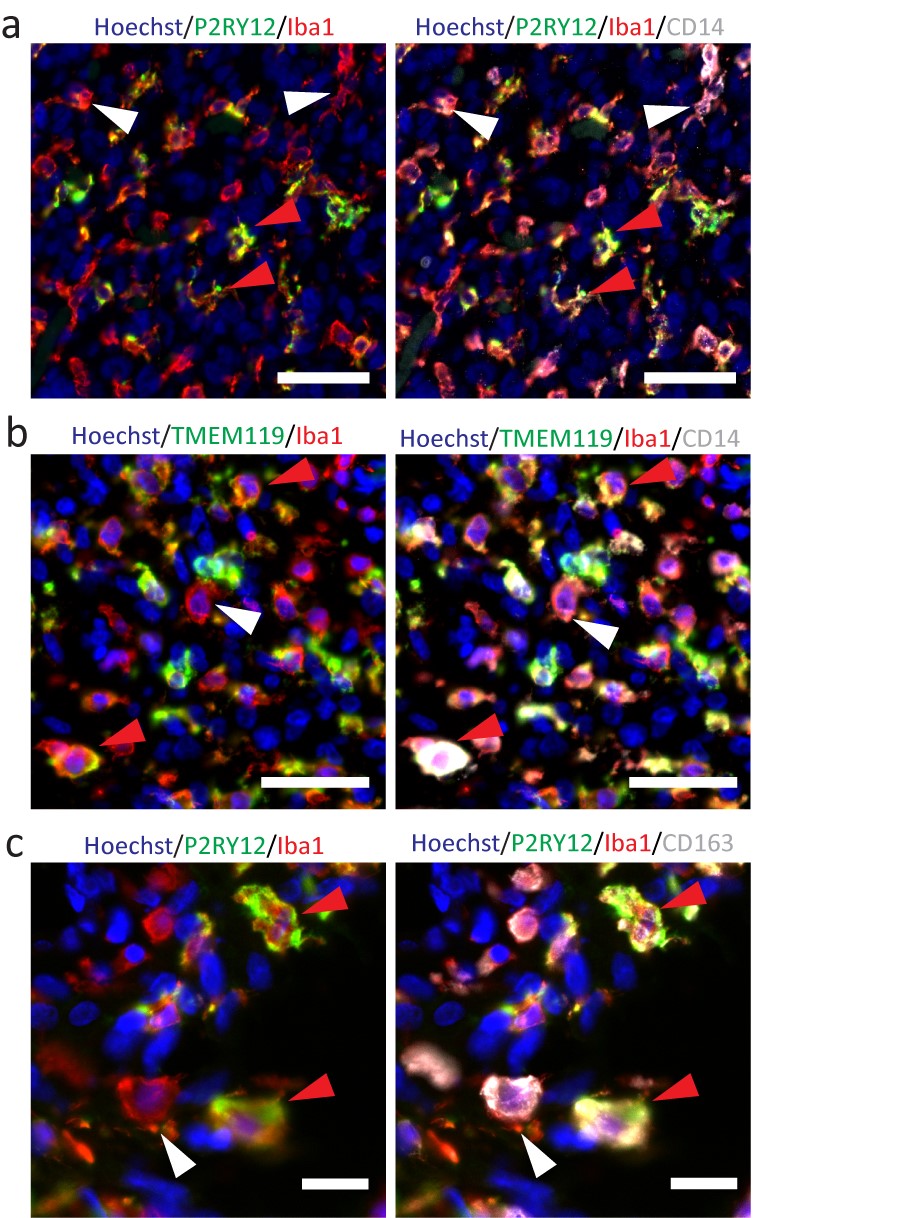

Supplement: vdab031_suppl_Supplementary_Figure_S1 [file vdab031_suppl_supplementary_figure_s1.jpeg]

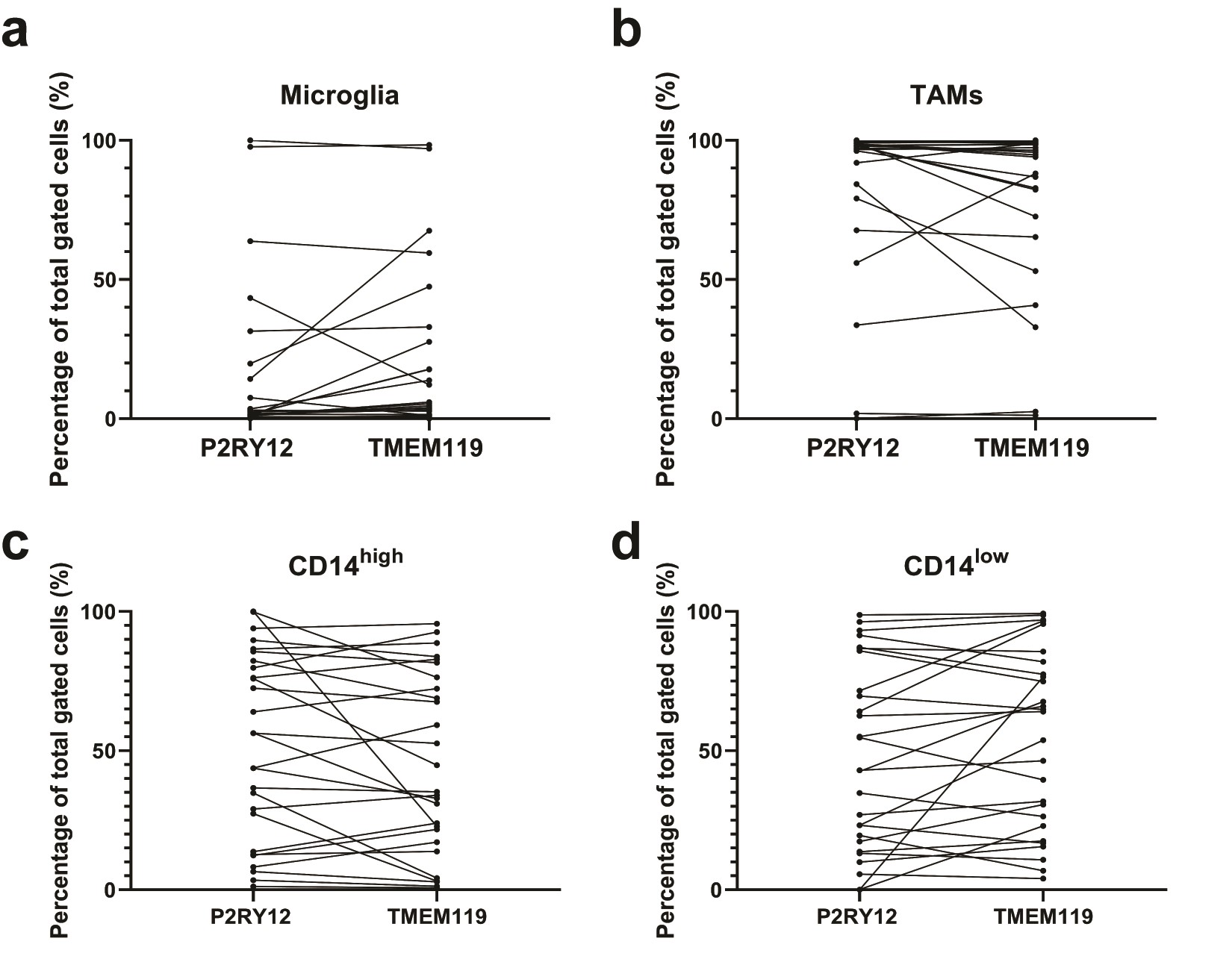

Supplement: vdab031_suppl_Supplementary_Figure_S2 [file vdab031_suppl_supplementary_figure_s2.jpeg]

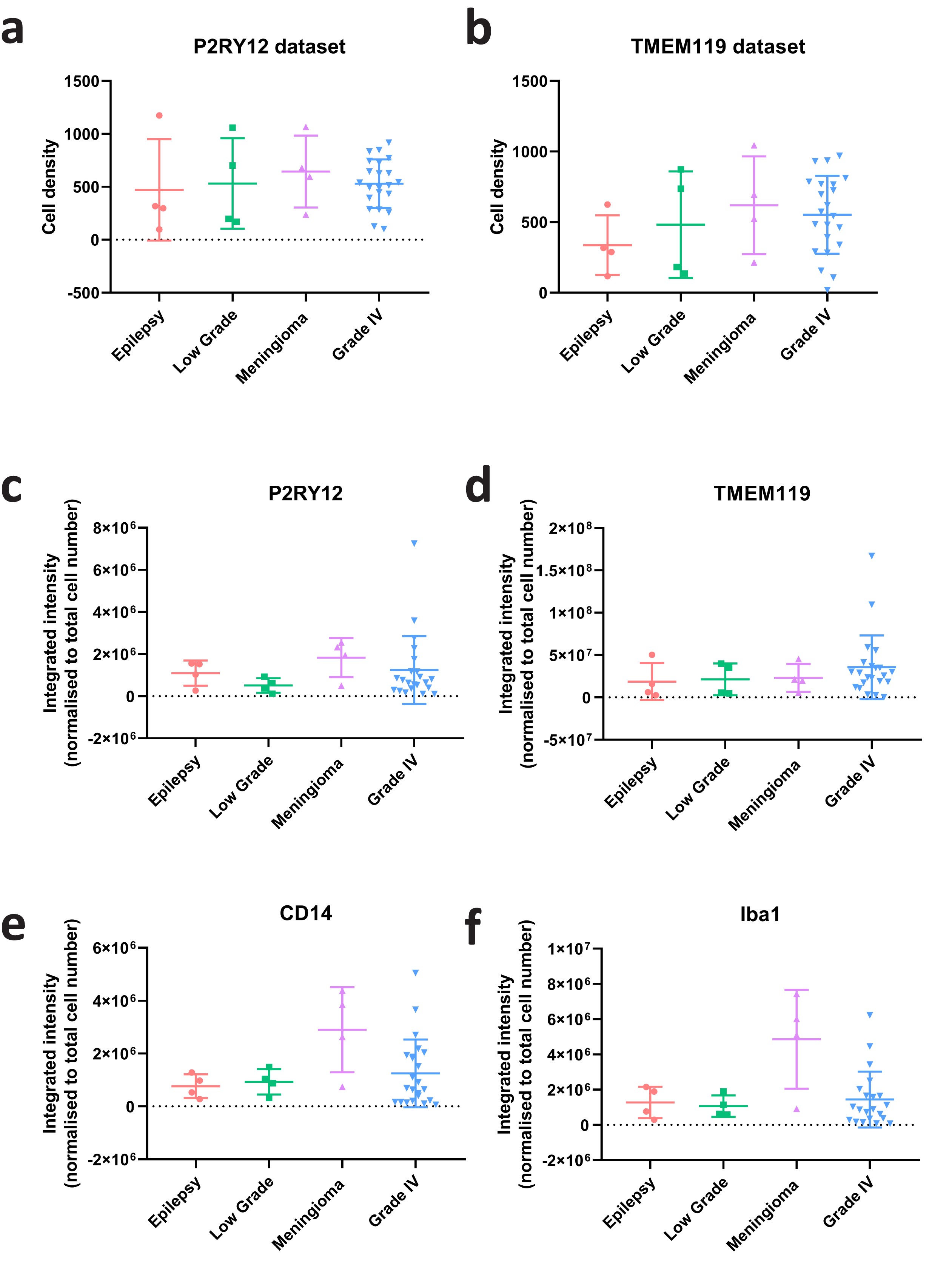

Supplement: vdab031_suppl_Supplementary_Figure_S3 [file vdab031_suppl_supplementary_figure_s3.jpeg]
