# Supplementary material for: Single-cell image analysis reveals a protective role for microglia in glioblastoma
Source: Neurooncol Adv. 2021 May 4;3(1):vdab031. doi: 10.1093/noajnl/vdab031 (PMC8284623; doi:10.1093/noajnl/vdab031)
Supplement: vdab031_suppl_Supplementary_Table_S1 [file vdab031_suppl_supplementary_table_s1.docx]

**Table S1. Summary of patient information from all cases used in immunohistochemical staining**

| Pathology | N | Average age | MGMT methylated | IDH1 MT |
| --- | --- | --- | --- | --- |
| Epilepsy | 7 | 38.6 | NA | NA |
| Glioblastoma (grade IV) | 24 | 60.6 | 9 | 1 |
| Low-grade (I-II) | 4 | 40 | NA | 1 |
| Meningioma (I) | 4 | 62 | NA | NA |
