# Supplementary material for: Single-cell image analysis reveals a protective role for microglia in glioblastoma
Source: Neurooncol Adv. 2021 May 4;3(1):vdab031. doi: 10.1093/noajnl/vdab031 (PMC8284623; doi:10.1093/noajnl/vdab031)
Supplement: vdab031_suppl_Supplementary_Table_S2 [file vdab031_suppl_supplementary_table_s2.docx]

**Table S2. List of applied primary and secondary antibodies**

| Primary antibody | Company, Catalogue number | Concentration | Visualisation | Reference |
| --- | --- | --- | --- | --- |
| Rabbit anti-P2RY12 | Sigma Aldrich, HPA014518 | 1:500 | TSA Alexa Fluor 488 | ^33^ |
| Rabbi anti-TMEM119 | Abcam, Ab185333 | 1:500 | TSA Alexa Fluor 488 | ^33,34^ |
| Goat anti-Iba1 | Abcam, Ab5076 | 1:1000 | Alexa Fluor 647 secondary | ^33,34^ |
| Mouse anti-CD14 | Abcam, Ab182032 | 1:500 | Alexa Fluor 594 secondary | ^35^ |
| Mouse anti-CD163 | Abcam, Ab201461 | 1:100 | Alexa Fluor 594 secondary | ^36^ |
