# Supplementary material for: Single-cell image analysis reveals a protective role for microglia in glioblastoma
Source: Neurooncol Adv. 2021 May 4;3(1):vdab031. doi: 10.1093/noajnl/vdab031 (PMC8284623; doi:10.1093/noajnl/vdab031)
Supplement: vdab031_suppl_Supplementary_Table_S3 [file vdab031_suppl_supplementary_table_s3.docx]

**Table S3. Summary of statistical analysis of population proportions**

| Tissue subtype | Population ratio | N of cases | Proportions (%) | SD (%) |
| --- | --- | --- | --- | --- |
| Epilepsy | Microglia to TAM ratio (P2RY12 immunoreactivity) | 4 | 92.5 : 6.9 | 7.9, 7.5 |
|  | Microglia to TAM ratio (TMEM119 immunoreactivity) | 3 | 97.8 :1.7 | 0.5, 0.6 |
|  | CD14 ^high^ to CD14 ^low^ ratio | 5 | 4.6 : 95.3 | 5.4, 5.6 |
|  | CD163 ^high^ to CD163 ^low^ ratio | 5 | 2.4 : 97.9 | 0.4, 0.4 |
| Low-grade | Microglia to TAM ratio (P2RY12 immunoreactivity) | 4 | 1.8 : 97.9 | 0.8, 0.8 |
|  | Microglia to TAM ratio (TMEM119 immunoreactivity) | 3 | 2.1 : 97.8 | 1.1, 1.2 |
|  | CD14 ^high^ to CD14 ^low^ ratio | 4 | 38.3 : 60.9 | 30.1, 30.9 |
| Meningioma | Microglia to TAM ratio (P2RY12 immunoreactivity) | 4 | 1.0 : 98.8 | 0.9, 1.0 |
|  | Microglia to TAM ratio (TMEM119 immunoreactivity) | 6 | 7.4 : 92.4 | 7.2, 7.8 |
|  | CD14 ^high^ to CD14 ^low^ ratio | 6 | 62.0 : 37.4 | 33.8, 33.7 |
| Grade IV | Microglia to TAM ratio (P2RY12 immunoreactivity) | 22 | 11.5 : 87.9 | 16.7, 17.3 |
|  | Microglia to TAM ratio (TMEM119 immunoreactivity) | 24 | 20.1 : 79.9 | 23.2, 23.3 |
|  | CD14 ^high^ to CD14 ^low^ ratio | 29 | 48.4 : 52.7 | 32.6, 31.7 |
|  | CD163 ^high^ to CD63 ^low^ ratio | 15 | 76.6 : 23.4 | 27.1, 27.2 |
