# Supplementary material for: Single-cell image analysis reveals a protective role for microglia in glioblastoma
Source: Neurooncol Adv. 2021 May 4;3(1):vdab031. doi: 10.1093/noajnl/vdab031 (PMC8284623; doi:10.1093/noajnl/vdab031)
Supplement: vdab031_suppl_Supplementary_Materials [file vdab031_suppl_supplementary_materials.docx]

**Figure S1. CD14 and CD163 are expressed by both microglia and TAMs in human glioblastoma tumour tissue.**

Representative images of fluorescent triple labelling of Iba1 with P2RY12 (a) or TMEM119 (b) and CD14, or P2RY12, Iba1 and CD163 in tumour tissue (c). Red arrows indicate Iba1^+^ P2RY12^+^ or TMEM119^+^ microglia, white arrows indicate Iba1^+^ only TAMs. Scale bar = 50 µm.

**Figure S2. Percentage of gated microglia and TAMs and CD14 ^high/low^ cells are consistent across both P2RY12 and TMEM119 staining and analysis runs.**

Percentage of gated microglia (a) and TAMs (b) in both P2RY12 and TMEM119 datasets across paired cases. Percentage of gated CD14 ^high^ (c) and CD14 ^low^ (d) cells in both P2RY12 and TMEM119 datasets across paired cases (Paired T-test, *P > 0.05*).

**Figure S3. Global expression of P2RY12, TMEM119 and Iba1 stable across brain tissue subtypes.**

Cell density (total gated cells/mm^2^) across tissue subtypes in each dataset (a, b). Global expression (integrated intensity of marker per mm^2^/total gated cells) of P2RY12 (a), TMEM119 (b), CD14 (c) and Iba1 (c) across epilepsy, low-grade tumour, meningioma and grade IV tumour tissue. Kruskal-Wallis with Dunns multiple comparison test. Data presented as mean ± SD, P > 0.05.
